# Supplementary figures and images for: Reward Bases: A simple mechanism for adaptive acquisition of multiple reward types
Source: PLoS Comput Biol. 2024 Nov 19;20(11):e1012580. doi: 10.1371/journal.pcbi.1012580 (PMC11614280; doi:10.1371/journal.pcbi.1012580)

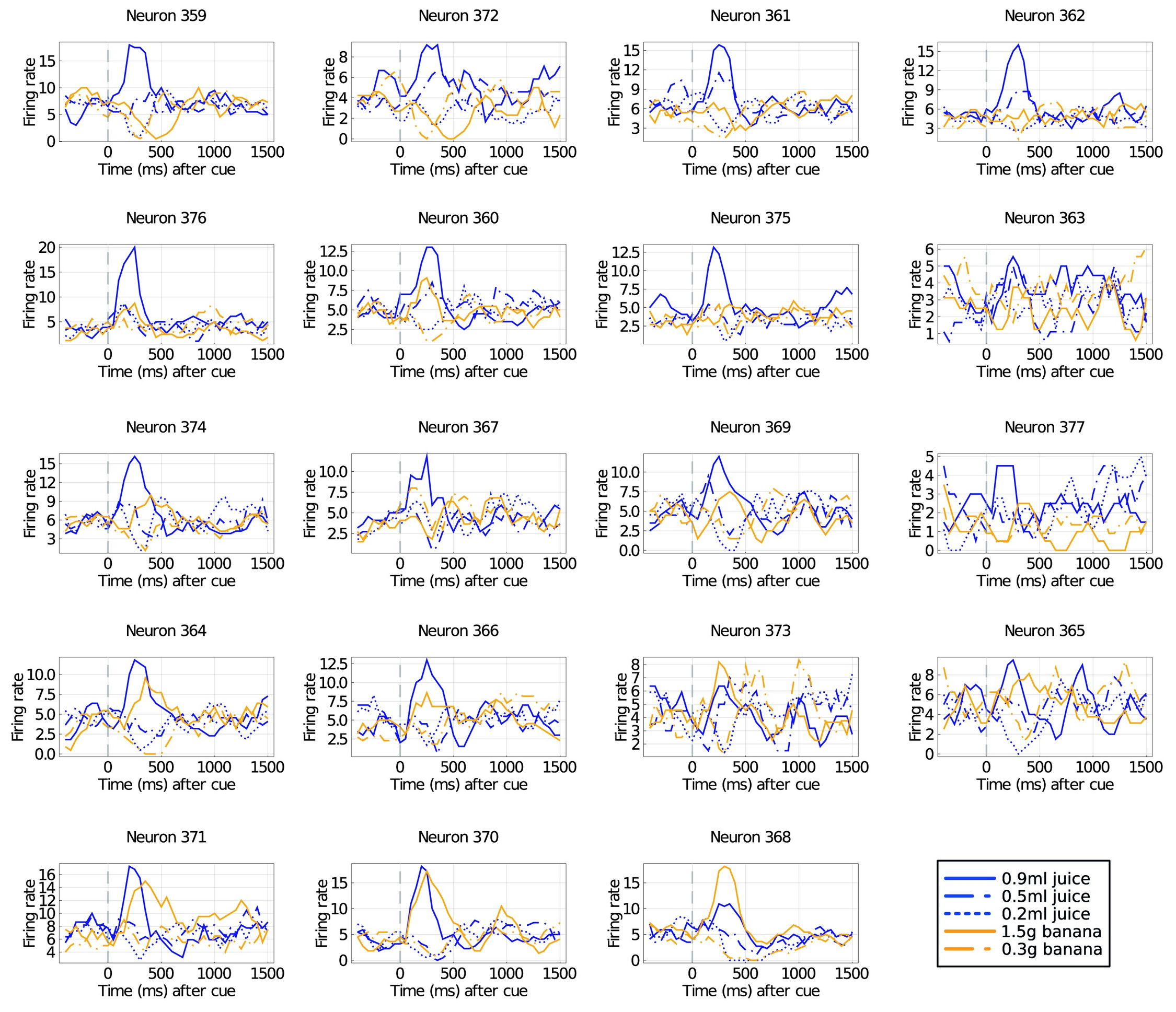

Supplement: S1 Fig — The plot shows the firing rate as a function of time within a trial (see Methods Section 4.2), where time 0 corresponds to onset of the stimulus indicating which reward type will be presented. The neurons are ordered by the interaction coefficient of value and identity (i.e., as in Fig 4C). (TIF) [file pcbi.1012580.s001.tif]

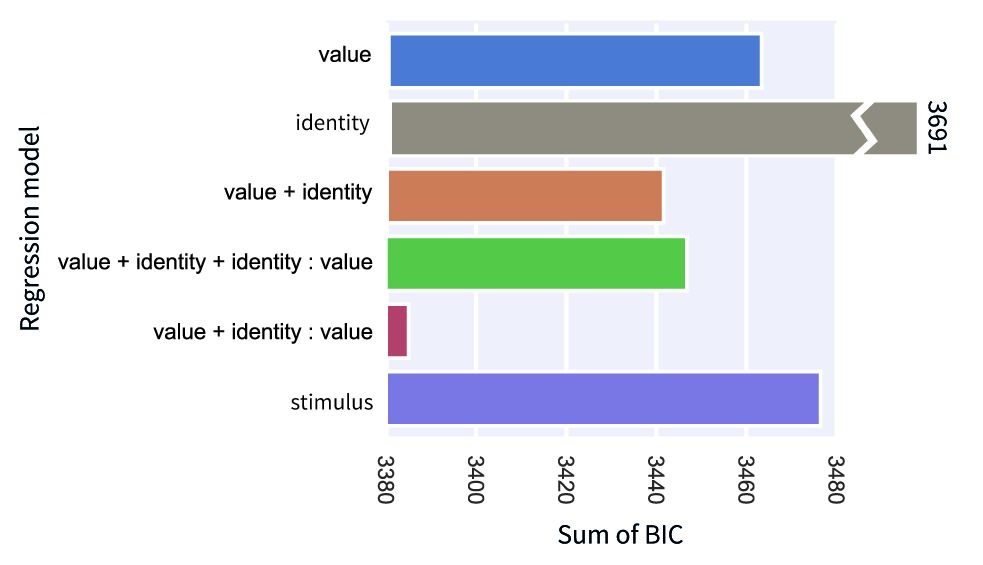

Supplement: S2 Fig — The graph shows results of an analysis analogous to that in Fig 4B, but here Bayesian Information Criterion (BIC) is measured and reported. (TIF) [file pcbi.1012580.s002.tif]

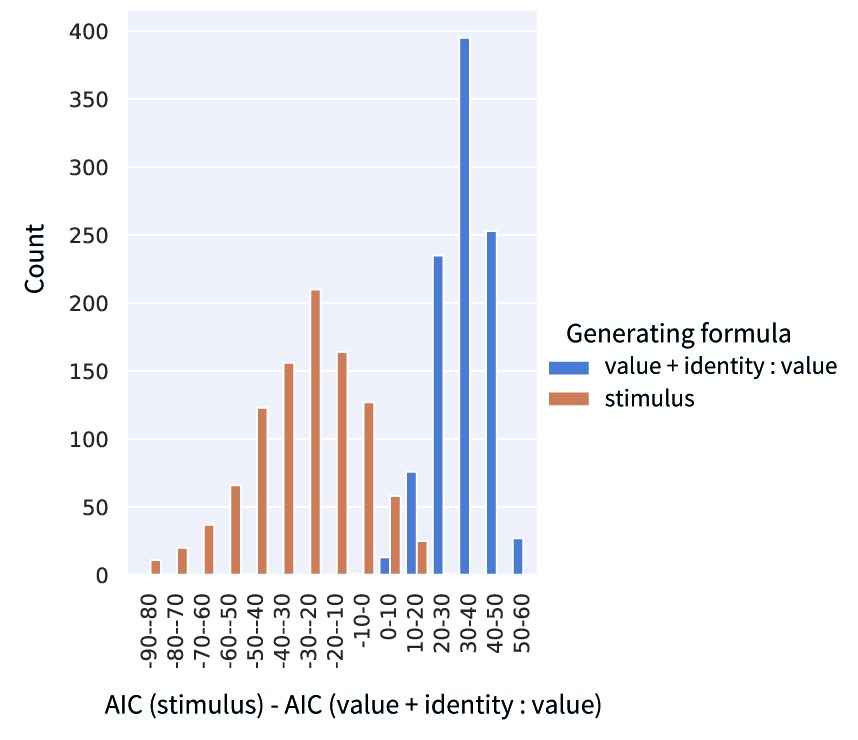

Supplement: S3 Fig — For each model, we generated 1000 surrogate datasets, each containing surrogate activity of the same number of neurons on the same number of trials as in the experiment. To obtain surrogate data, we generated for each trial a prediction with the two fitted models. When generating the prediction, we also added a random number from a normal distribution with the standard deviation equal to the standard deviation of the residuals obtained when we fitting the model to the data from a given neuron. We then fitted both formulas to the data generated from both models, and summed the AIC score across neurons. We plot the results as a histogram of the difference of AIC score between the fit with formula “stimulus” and the fit with formula “value + identity:value”. Different colours indicate the model used to generate the surrogate data. There is little overlap between orange and blue histograms indicating that the AIC can reliably distinguish between the two corresponding models. The difference between the AIC scores computed from real data for the two models in Fig 4B was 16.8. Such or higher difference was obtained only for 0.8% of surrogate datasets generated from “stimulus” model, indicating that it is very unlikely for the actual data to be generated by that model. (TIF) [file pcbi.1012580.s003.tif]

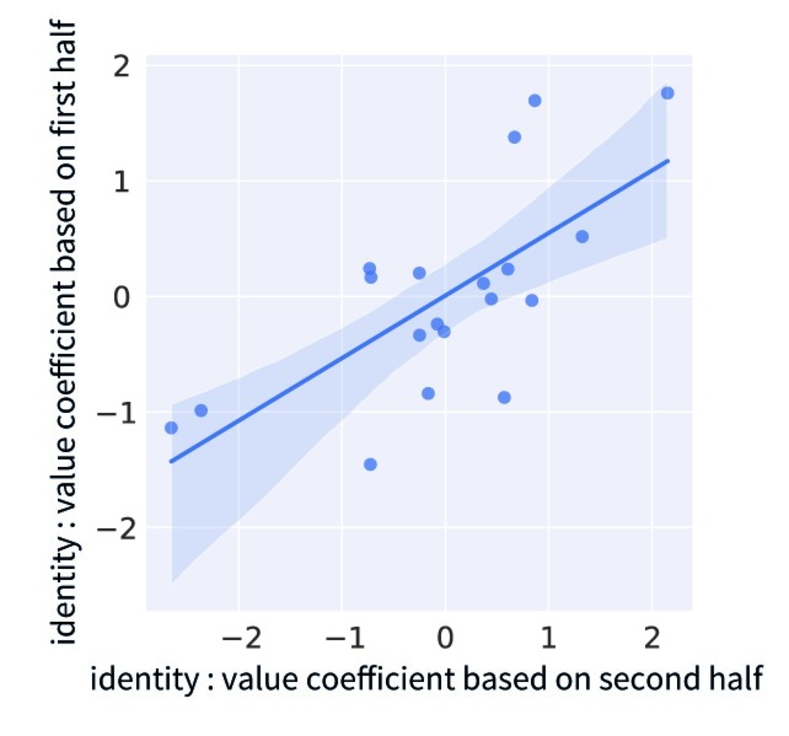

Supplement: S4 Fig — Here, the same analysis as in Fig 4C is conducted, i.e., the regression model “value + identity: value” is fitted to the data, but this is done separately for different portions of the data—first time based on the first half of the trials in the recording session, and second time using the second half of the trials. The interaction coefficients from the two fittings are plotted in a scatter graph, in which each dot corresponds to a neuron, and its x and y coordinates correspond to the coefficients. As can be seen, there is a significant correlation (r = 0.69, p = 0.001) between the coefficients from the two periods, indicating that the selectivity of neurons for different reward types is stable. The solid line represents the best-fit linear regression line. The shaded area around the regression line, indicates the 95% confidence interval. (TIF) [file pcbi.1012580.s004.tif]
