# Supplementary material for: Reward Bases: A simple mechanism for adaptive acquisition of multiple reward types
Source: PLoS Comput Biol. 2024 Nov 19;20(11):e1012580. doi: 10.1371/journal.pcbi.1012580 (PMC11614280; doi:10.1371/journal.pcbi.1012580)
Supplement: Attachment — Submitted filename: response.pdf [file pcbi.1012580.s005.pdf]

Response to the comments of the Reviewers

↪ We thank the Editor and the Reviewers for very useful feedback and suggestions. We addressed all of them, as explained in detail below, and we feel that they significantly improved the manuscript.

Reviewer 1

The main comment relates to prior literature. The theoretical idea is not new, and the authors should discuss in more details the specific theoretical contribution of their work beyond references 44-46.

↪ We added a paragraph discussing the contributions of the Reward Bases model beyond the homeostatic RL models:

> "··· *The Reward Bases model is related with the models of homeostatic regulation [44, 45, 46], which also consider multiple dimensions of rewards. However, in these models, the agent learns a single overall value function, rather than separate value bases associated with individual reward dimensions. Therefore, when the motivation changes, these models require experiencing reward to change the overall value function (analogously as the multi-objective RL model discussed above). The contribution of the Reward Bases model is that it describes how value can change without experiencing reward, and hence can capture the data on instant changes in behaviour and dopamine release due to changes in motivation.* ···" — *Page 17, Lines 515-521*

Another, more minor comment relates to the analysis of the experimental work of reference 43. The authors carefully acknowledge that the number of neurons that could be used for the analysis is very small (19) which makes it difficult to draw conclusions. Given the centrality of this experiment in this manuscript, I suggest that all neurons will be presented as a supplementary figure, and not only the examples of figure 3.

↪ We added a new supplementary S1 Figure showing activity of all recorded neurons:

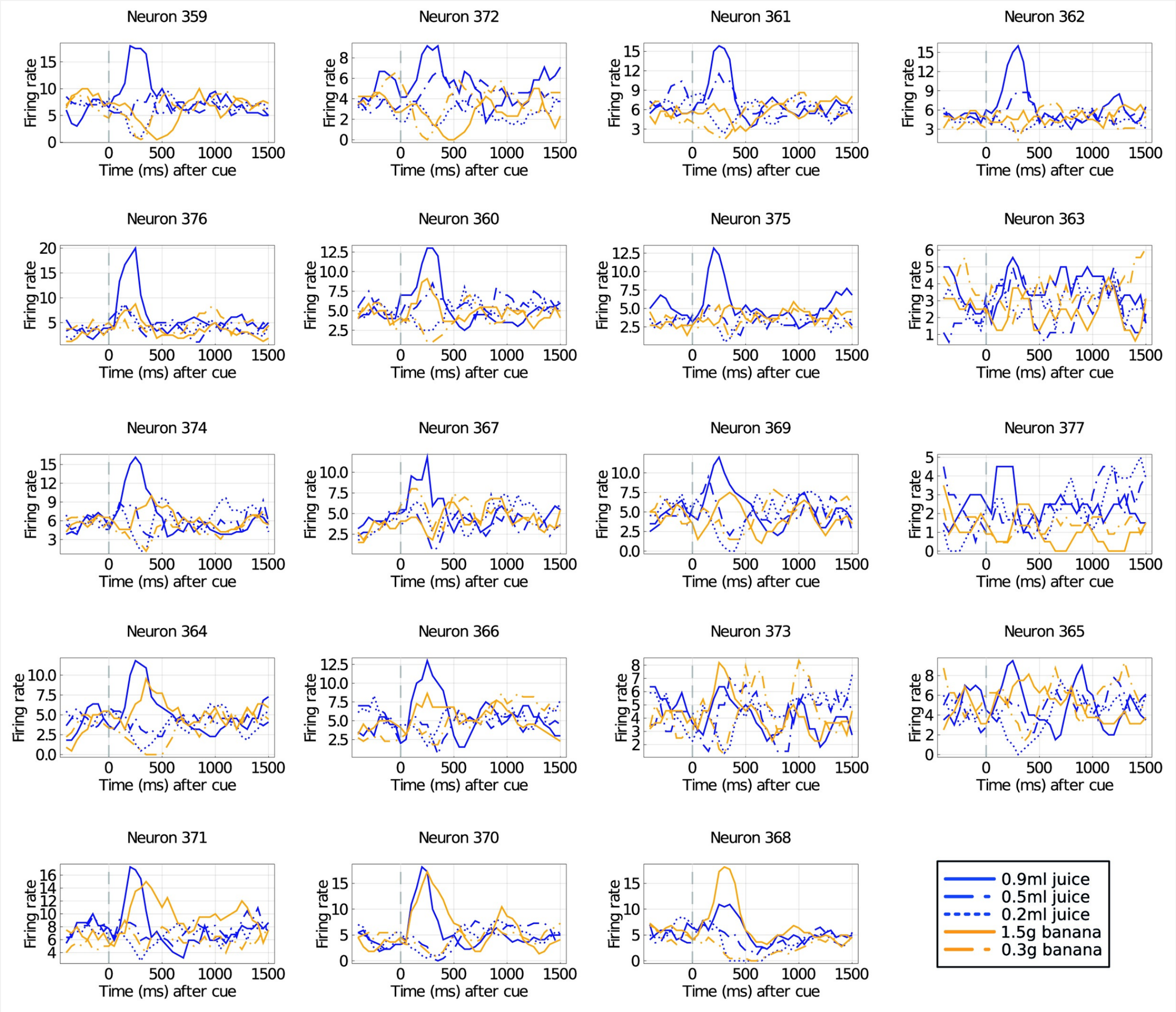

Another, even more minor point is that I find Figure 1 and the accompanying text to be trivial, and hence not very helpful. I recommend shortening the manuscript by removing it.

↪ We feel that Figure 1 is valuable for providing intuitions for the model, because when we presented it in various talks, people in the audience found it very helpful for understanding of the model. Furthermore, we employ the setup of Figure 1 in later simulations comparing Reward Bases with successor representation, so this setup needs to be introduced. Therefore, we prefer to keep Figure 1 in the manuscript.

Finally, there are many references to the Methods section. Given the large number of sections in the Methods section, it would be useful to refer the reader to the particular subsection of the Methods section.

↪ We have numbered all sections in the manuscript, and whenever we refer to a particular section of the Methods, we now provide a section number.

Reviewer 2

MAJOR COMMENTS

1. Data Analysis

The model comparison applied to the regression analysis used to validate the model with the first archival data set ends by selecting a model with a main effect of value and an interaction term for value and stimulus identity. However, this violates the hierarchical principle in regression, which requires all main effect terms for all factors used in the interaction term to be present in the model. Otherwise, the interaction term absorbs variance from the missing main effect term and impacts overall model performance, regardless of the statistical significance of the individual regression coefficients.

This is a major issue because the fact that this model, with the interaction term, was the best fit to the data is used as a validation of the predictions of the Reward Bases model. Based on the AIC values shown in Figure 4A, the stimulus alone model would be the best fit if the value + value*identity model were removed from the comparison set. If I read this correctly, that would not validate the theoretical model the authors have proposed. This seems important.

↪ We added a clarification that the model including main effect of value and its interaction with identity is equivalent to the model including the main effect of food prediction error and the main effect of juice prediction error, so it could be formulated without any interactions:

> "… *The model employing subjective value and its interaction with identity is also equivalent to a model assuming that dopamine neurons encode a combination of prediction errors associated with different reward bases, that we will introduce later (in this model dopamine is explained by the main effect of prediction error for food and the main effect of prediction error for drink).* …"
>
> — *Page 9, Lines 236-239*

Please note that Figure 4A also shows a comparison with a model including value, identity and their interaction, but this model does not provide as good description of the data.

Additionally, regarding data analysis, it is unclear how the permutation tests were run, whose results are plotted in Figures 3G and 4C. I am unsure as to what I am looking at in these plots and how they relate to the relevant null hypothesis test being performed.

↪ We clarified the null hypothesis and shuffling procedure in Figure 3G:

> "… *We then compared this result with the correlation expected from a null hypothesis that the responses of dopamine neurons are the same after the stimuli predicting the largest amount of banana and juice. To obtain a null distribution, we recalculated the correlation 1000 times: In each iteration we randomly separated trials with stimuli predicting the largest amount of banana and juice into two groups, calculated the averaged response magnitude of each trial group for each neuron, and then calculated a correlation between responses on the two trial groups across neurons. The distribution of such correlations is shown in Figure 3G, and in all 1000 repetitions the correlation obtained from the shuffled data was larger than from the experiment, suggesting that the experimental correlation is significantly lower than expected from the above null hypothesis ($p<0.001$).* …"
>
> — *Page 8, Lines 216-224*

We also added analogous clarification for Figure 4C:

> "… *To investigate if this number of significant interaction coefficients could arise by chance, we compared it with the number expected from a null hypothesis that the responses of dopamine neurons do not depend on the type of reward predicted by the stimulus. To obtain a null distribution we repeated the following analysis 1000 times: In each iterations we shuffled assignments of trials to the reward type (i.e., we replaced the identity regressor across all trials recorded for a given neuron by its random permutation), performed regression predicting dopamine response from value and its interaction with (shuffled) identity, and counted the number of neurons with significant interaction coefficient. Figure 4C shows that in all 1000 repetitions the observed number of neurons with significant interaction coefficient was smaller than in the experimental data, so the probability of obtaining 7 significant neurons under the null hypothesis is* $p < 0.001$. …"
> — *Page 9, Lines 259-268*

2. Logic Structure

One major, but not critical, issue with the logic structure is that the evaluations of the model seem to start with the most complex cases (representations of dopamine neurons) and then move to the simplest (demonstration of how physiological states impact behavior). This seems odd. Wouldn't it make sense to start by showing the simple fact that the proposed model can capture a classic behavioral effect (i.e., choice behavior with altered physiological states described in the "Behavior reflects instant revaluation in novel physiological state" section) and then demonstrate that the representation of value in neurons matches the mixed selectivity predicted by the model?

↪ In the initial version of the manuscript, we indeed presented simulation of behaviour at the start, but then changed the order, because the analyses of responses of dopamine neurons are most surprising, hence we feel the readers may prefer to find about them first.

3. Model Comparisons

As I was reading this, I kept thinking about how similar this model appears to be to distributional RL. The authors correctly state that both successor representation and distributional RL are quite similar in structure but different in both form and predictions than the current proposed model (see Discussion). However, the only direct comparison in the paper is done with successor representation (and simple TD learning, which is obviously going to be ineffective). So, I am curious why the authors chose to exclude the other class of learning models most similar to the Reward-Based model.

↪ We added a sentence explaining why we do not compare the performance of distributional RL:

> "… *Since distributional RL does not learn reward associated with different reward dimensions, it cannot explain changes in behaviour and dopamine response after change in motivation.* …"
> — *Page 17, Lines 540-541*

MINOR COMMENTS

- Pg 1, Abstract: "recorded after stimuli predicting distinct types of rewards" is a sentence fragment. I believe something like "were delivered" should be at the end.

- Pg 2, Line 43: “for controlling behaviour” is a typo. It should be “to control behaviour.”
- Pg 11, Line 268: “coefficient” should be “coefficients.”
- Pg 11, Line 280: “with a dopamine neurons” should be “with dopamine neurons.”

↪ The typos listed above have been corrected.

- Pg 11, Lines 280-299: The “Learning with mixed selectivity” section feels very rushed. This needs to be elaborated on a bit more.

↪ We extended the description of the implementation of the model:

> “... *This computation could arise from the connectivity pattern shown in Figure 2C, where each dopamine neuron k receives inputs from reward and striatal neurons associated with distinct reward bases i through connections with weights* β_i^k. *The value bases are then learned in this model through the standard TD error rule using weighted sums of the prediction errors, which could arise if dopamine neuron k also sends the prediction error to the stiatal neurons encoding value bases i through connections with the same weights* β_i^k. ...” — *Page 10, Lines 298-302*

We also extended the description of simulation results:

> “... *For example, the left display shows the estimated values in the food dimension for the five stimuli. As learning progresses, the estimated values converge to the true subjective values for the stimuli predicting banana, while they converge to 0 for stimuli predicting juice. Interestingly, the value in food dimension initially increases for stimuli predicting juice, because the striatal neurons representing food value receive a mixture of prediction errors, but then it correctly decays to 0.* ...” — *Page 11, Lines 307-311*

- Pg 13, Line 320: “more of food” should be “more food.”

↪ The above typo has been corrected.

Reviewer 3

Major comments: The use of the term “state” in the paper is somewhat equivocal. For example, in lines 7-8 the authors write that a simple neural circuit “learns the availability of different reward types in different states, and then combines them according to the motivational state to control behavior.” (my emphasis). In line 13 they refer to “cortical states” and in line 23 to rats “placed in a physiological state”. In lines 71-72 they describe the agent’s state in a technical sense of Reinforcement Learning (RL) models, as something that “may correspond to an animal’s location in space or a stimulus presented during an experiment”. It would be useful to delineate and clarify the relationship between the different uses – technical and colloquial – of the term “state”.

↪ We added a sentence to the first paragraph of the paper clarifying that:

"··· *Throughout this paper we distinguish between two aspects of animal's state: physiological state such as hunger or thirst, and environmental state such as animal's location in space or a stimulus presented during an experiment.* ···" — *Page 2, Lines 18-20*

To clarify this distinction, throughout the whole manuscript we ensured that word 'state' never occurs alone but is preceded either by 'physiological' or 'environmental'. We also replaced phrase 'cortical state' by 'environmental state'.

Furthermore, states in RL are supposed to be summaries of the relevant features of experience for guiding goal-directed learning and behavior. Shouldn't then motivational or physiological "states" also be considered as part of the RL state?

↪ We have considered a model with RL state including both the environmental state and physiological state, and made there description of this idea clearer by adding appropriate adjectives before word 'state':

"··· *Finally, another possibility for extending model-free TD learning methods to handle varying reward functions is to simply assume that x includes both environmental and physiological state, and use RL to estimate the value function of such combined state.* ···" — *Page 18, Lines 571-573*

We have also extended our discussion of why such model may not adapt to changes in environment as quickly as Reward Bases:

"··· *This method, however, suffers from three substantial disadvantages compared to the Reward Bases approach described in this paper. Firstly, such a model does not incorporate any prior knowledge on how the physiological state affects the value function, so it would need to learn this dependence from training data. Hence training of such a model would take much longer than Reward Bases which already assumes the dependence (Equation 5). Secondly, it entails a substantial increase in the ultimate size of the state-space which hampers the generalizability and sample-efficiency of the resulting algorithm, and is especially acute in the presence of continuously-valued physiological states which would require the animal to essentially learn its value functions from scratch for every possible setting of the physiological state. Continuously varying physiological states are handled naturally, however, in the Reward Bases model by just using the weighting coefficients. Thirdly, this approach of extending the state-space is not guaranteed to achieve the zero-shot generalisation that the Reward Bases scheme is capable of. This is because it uses standard TD learning which can only associate outcomes with a novel physiological state by directly experiencing those outcomes. For instance, in the salt-deprivation experiments [10], the combined state method may not exhibit instant generalisation but may have to experience several positive pairings of the salt with the salt-deprived physiological state to allow for this generalisation – while in the original experiment the rats were specifically never previously salt-deprived in their lives to prevent this possible association developing outside the experimental paradigm.* ···" — *Page 18, Lines 573-589*

On a related point, the authors mention (lines 50-52) that dopamine neurons encode information about diverse variables (which are not directly reward related) such as movement and sensory features. In other words, dopamine neurons seem to encode state information, not only reward prediction error, as posited by TD learning algorithms, regardless of whether reward is thought of a scalar or multi dimensional. It would be interesting to know how this "state-representational" aspect of dopamine activity can be interpreted in light of their model.

↪ We have previously included a paragraph discussing that the diverse responses of dopaminergic neurons have been interpreted as errors in different predictions. This paragraph also discusses how the Reward Bases model fits into this hypothesis. We copy this paragraph below:

> "··· *Our results add support to a general theory that the striatum is parcellated into modules predicting different quantities, and the dopamine neurons projecting to a given parcel of the striatum compute the error in prediction made by this parcel [60]. In the case of Reward Bases model, different parcels located in ventral striatum compute different value bases, but other striatal regions may compute different quantities, and consequently corresponding dopamine neurons may encode errors in different predictions. For example, dopamine neurons in the tail of the striatum were proposed to encode prediction errors specifically for aversive stimuli [61, 62], or action prediction errors [63]. Moreover, dopamine neurons have been shown to encode errors in predictions of sensory features of reward in addition to value [36]. There is also growing evidence that dopamine neurons are sensitive to various aspects of locomotion and kinematic behaviour [35, 64, 65, 66] as well as choice behaviour [67, 68] and indeed that this heterogeneity is topographically organised due to spatially organised cortical projections [66, 34].* ···" *— Page 15, Lines 458-468*

How is the lack of correlation between individual neuron responses to juice and banana (Fig 3G) reconciled with the fact that most neurons exhibited mixed selectivity to both reward types (Fig 5A)?

↪ We modified the description of results in Figure 5A to clarify that some neurons were selective for a single reward dimension:

> "··· *Although there are some neurons with coefficients for one dimension close to 0, overall the points are spread out suggesting that the majority of dopamine neurons encode different mixtures of prediction errors.* ···" *— Page 10, Lines 283-285*

The neurons encoding just the reward prediction error for juice produce negative responses after stimulus predicting the largest amount of banana, resulting in the lack of correlation in Figures 3F-G.

The simulation demonstrating the physiological state dependent reevaluation aspect of the model felt a bit underwhelming. It shows that adding a state dependent parameter on the reward modulates state dependent approach behavior (by the way, I am not sure how approach behavior is defined, this should be explained in the caption or text explaining figure 7). It would be nice to see a less trivial demonstration of this important aspect of their model.

↪ We added an explanation for why we feel this task provides the clearest demonstration of the difference between the models in accounting for effects of revaluation seen in behaviour:

> "· · · *Although this simulated task is very simple, we choose to present it, because we feel that due its low complexity it best illustrates the difference between the models, and it is striking that the TD learning cannot account for behaviour in so simple task.* · · · " — *Page 14, Lines 406-408*

We have also added a sentence in the caption of Figure 7 explaining how approach behaviour is defined:

"In panels B and C, 'Approach behaviour' is equal to the estimated value of environmental states scaled by a constant chosen to match these values to the experimental data, as described Methods Section 4.6."

The authors should include more detailed comparisons between their model and related ones such as feature weighted reinforcement learning, e.g., in Niv et al. 2015, the feature-specific reward prediction error model of Lee et al. 2022 or the multithreaded TDRL model of Takahashi et al 2023. More generally, it might be useful to compare the reward bases model with other theoretical frameworks coupling the representational and evaluative aspects of goal-directed learning, e.g., Amir et al 2023.

↪ We thank the Reviewer for pointing us to these highly relevant papers we were not aware of. We added discussion of the studies by Niv et al. and Lee et al.:

> "· · · *The Reward Bases model is also related to a model of RL in multidimensional environment [83]. This study demonstrated that if participants are asked to learn reward probability associated with stimuli described by multiple features, their behaviour is best described by a model assuming that they learn values associated with the individual features, and compute the value of a stimulus as a sum of values of individual features. Although the formulation of this model is different (e.g., it only employs a single prediction error), this study suggests that decomposing value into multiple dimensions is a general feature of biological RL.*
>
> *The Reward Bases model is also related to a feature specific prediction error model [84]. It explains the diversity of responses of dopamine neurons, by assuming that, due to anatomical constraints, individual dopamine neurons receive inputs from striatal neurons representing value of just a subset of features environmental states. So in this respect that model is similar to Reward Bases. However, that model assumes that as a population, dopamine neurons encode the standard prediction error, and this single prediction error is used to update a single value function represented in the striatum, hence it differs from the Reward Bases which assumes the basal ganglia learn multiple value functions and flexibly combine them according to the physiological state.* · · · " — *Page 17, Lines 522-534*

We rewrote the discussion of the model by Takahashi et al. as on rereading this paper, we realised that this models shares key computational similarities with the model of Huang and Grabenhorst, so we reviewed these two models together:

> "··· *Our results are consistent with studies showing that primate and rodent behaviour is well described by models that learn expected reward in multiple dimensions via prediction errors for different reward dimensions [80, 81].* ···" — *Page 16, Lines 494-496*

We also added a reference to the work of Amir et al. while discussing learning of novel reward bases:

> "··· *Computationally learning such bases could be achieved through finding the representations that are optimal for satisfaction of animal's needs [88].* ···" — *Page 19, Lines 622-623*

Line 620 ff. It would be helpful to motivate the construction of this model in Eq. 13, especially as later it is claimed to be equivalent to that of Eq. 12.

↪ Before introducing Equation 13, we added a sentence motivating it, and already highlighting its close relation with Equation 12:

> "··· *The model of Equation 12, can be rewritten in an equivalent form that is more directly related to the mixed selectivity implementation of the Reward Bases model. That equivalent regression model predicts* ···" — *Page 20, Lines 668-669*

If they are equivalent, why not just use one?

↪ We added a clarification for why both models are presented in the manuscript:

> "··· *Although these two regression models are equivalent, we present both of them in the paper, because they are both useful for different purposes: The one including value and its interaction with identity provides a natural comparison with other statistical models with regressors based on value and identity, while the regression model including prediction errors for food and drink is more closely related with the mixed selectivity implementation of Reward Bases.* ···" — *Page 9, Lines 239-243*

Also, it might be interesting to compare the linear regression model with more realistic (nonlinear) firing rate models.

↪ Please note the current regression models already assume non-linear dependence of dopamine activity on volume of rewards, because they are based on subjective value which is a non-linear function of the volume (Figure 3B). We feel that introducing further non-linearity is beyond the scope of this manuscript that already contains large number of models.

The authors include error bars on some of their simulation results (Fig 6C and Fig. 8 B-C). However, wouldn't the size of the error bars simply reduce with the number of simulation runs (3 in Fig. 6 and 10 in Fig. 8), and if so what is the meaning of including the bars (and how was the number of simulation runs chosen)?

↪ The Reviewer is correct that the error bars in simulations can be made arbitrarily small by increasing the number of iterations. However, we feel it is still useful to include the error bars to demonstrate that sufficient number of iterations was used. We added an explanation for how the number of iterations was chosen in the caption of Figure 6:

"(such small number of repetitions was sufficient as it already resulted in smaller error bars in simulation than in data)"

Fig. 4A, what would the AIC be for a model with only identity as a regressor?

↪ We have added a display of AIC for a model with only identity regressor in Figure 4A:

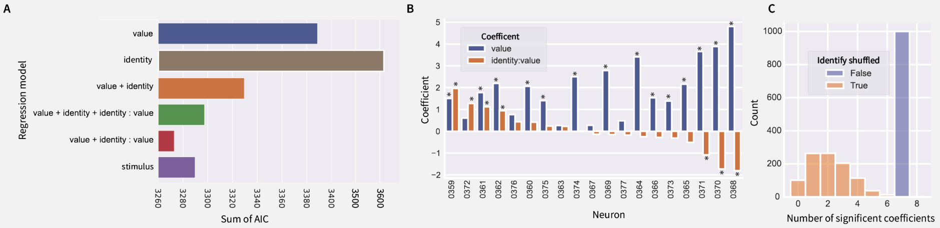

It would help to provide a comparison of the different model variants in one place.

↪ We added a new Table 1 summarising all model variants and their parameters:

Model	Prediction error	Value update	Evaluation	Parameters
Reward Bases	$\delta_i = r_i(x) + \gamma \mathcal{V}_i(x') - \mathcal{V}_i(x)$	$\Delta \mathcal{V}_i(x) = \alpha \delta_i$	$\mathcal{V}(x) = \sum_i m_i \mathcal{V}_i(x)$	α, m_i
Mixed selectivity	$\delta^k = \sum_i \beta_i^k \delta_i$	$\Delta \mathcal{V}_i(x) = \alpha \sum_k \beta_i^k \delta^k$	$\mathcal{V}(x) = \sum_i m_i \mathcal{V}_i(x)$	α, m_i, β_i^k
State-modulated	$\tilde{\delta}_i = m_i \delta_i$	$\Delta \mathcal{V}_i(x) = \alpha m_i \tilde{\delta}_i$	$\mathcal{V}(x) = \sum_i m_i \mathcal{V}_i(x)$	α, m_i

Specifically, the mixed selectivity and model and the physiological state-dependent model are interpreted as different but the way in which the dopaminergic response is fitted to the total prediction error in each one (Eqs. 15 & 23) seems mathematically equivalent, with m_i playing an analogous role to β_i. It would be good to make this equivalence clear, or explain if there is some essential difference between, other than that the parameters are interpreted differently, e.g., as neuronal selectivity in the first case and motivational weight in the second (or something similar).

↪ We clarified the difference in constraining various parameters in the caption of the new table: "Summary of variants of the Reward Bases model. The parameters of the models can be fit to experimental data, but different parameters are constrained in different ways: Motivation m_i is assumed to differ between experimental conditions in which animals have different physiological states. By contrast, coefficients β_i^k of the Mixed selectivity model can only be fitted to data from individual neurons, and are assumed to have different values for different neurons k."

Minor comments:

In the second to last row of tha abstract, "new physiological state" should be "new physiological states".

Line 13: "large amount" should be "large amounts".

Line 43: "for controlling" should be "to control".

Line 81: "seek" should be "seeks".

↪ We corrected the typos listed above.

Line 210: the authors claim that "multiple neurons decreased their firing", however according to figure 3F only two neurons show significantly decreased firing rates.

↪ This sentence has been modified to:

> "… *By contrast, dopamine neurons produced diverse responses, and two neurons even substantially decreased their firing rates after the stimulus predicting 1.5g of banana.* …" — *Page 8, Lines 212-214*

Lines 245-8: the text refers to grey, blue and yellow bars but the figure only seems to have blue and orange bars.

↪ We thank the Reviewer for spotting this typo (the text referred to colours in a previous version of the figure). We updated this text to match the current colours of the figure:

> "… *Blue bars show the coefficients for value, which are all positive, implying that the activity of all neurons is affected by the value. The orange bars show the coefficients of interaction between identity and value. A positive coefficient indicates that the corresponding neuron is more modulated by value for juice, while a negative coefficient indicates larger modulation for banana.* …" — *Page 9, Lines 254-258*

Line 440: what does it mean "rewards are more distinct", i.e., in what sense are they more distinct?

↪ As mentioned above, the discussion of the model by Takahashi et al. has been we completely rewritten, so the statement cited above is not longer present in the manuscript.

Line 488: what do the ellipses in the equation stand for?

↪ We do not see ellipses in our version of the manuscript that was submitted, and we wonder if there was a problem with displaying a pdf or a corrupted file.

Lines 533-534 the description of the extended state-space and how it similarly extends TD learning was not completely clear.

↪ As mentioned above, we have clarified these lines, by making reference to different types of state more explicit:

> "… *Finally, another possibility for extending model-free TD learning methods to handle varying reward functions is to simply assume that x includes both environmental and physiological state, and use RL to estimate the value function of such combined state.* …" — *Page 18, Lines 571-573*

Figure 5B is not so clear. In particular, all horizontal lines are dashed while according to the legend the higher valued ones should be solid.

↪ We have changed the pattern of horizontal lines in Figure 5B to match the caption:

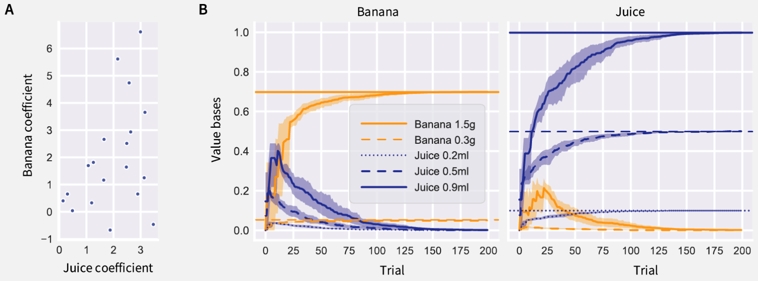

The description of the experimental paradigm in Fig. 6 was a bit unclear. The text says that animals were presented with a cue indicating which of the levers trigger reward delivery but the figure seems to show two cues and subsequently two levers extended (presumably representing a forced choice trial?).

↪ We have clarified task description in the caption of Figure 6:

" At the onset of forced trials, animals were presented with a single cue indicating which of the levers trigger reward delivery, while at the onset of choice trials both cues were shown. After a delay of 5s a single lever extends in the forced trials, or two levers extend in the choice trials (as shown in the figure). "

Some of the references seem to contain some scrambled or omitted text (e.g. [1])

↪ We have corrected reference 1 and checked other references.
