# Supplementary material for: Reward Bases: A simple mechanism for adaptive acquisition of multiple reward types
Source: PLoS Comput Biol. 2024 Nov 19;20(11):e1012580. doi: 10.1371/journal.pcbi.1012580 (PMC11614280; doi:10.1371/journal.pcbi.1012580)
Supplement: Attachment — Submitted filename: response.pdf [file pcbi.1012580.s006.pdf]

Response to the comments of the Editor and Reviewers

↪ We thank the Editor and the Reviewers for very useful feedback and suggestions. We addressed all of them, as explained in detail below, and we feel that they significantly improved the manuscript.

Editor

The mechanism for signaling internal states to the DA circuitry requires some additional justification, as does the immediacy of the changes in behavior that result.

↪ We refined the description of the implementation of the model the in striato-dopaminergic circuit, to make explicit how the dopaminergic activity could be modulated by physiological state to result in the immediate changes in behaviour. The refined model is summarised in Section 2.5, and the details of the model are described in Methods Section 4.4. We have also modified the figure showing the network implementation of the model and its simulations:

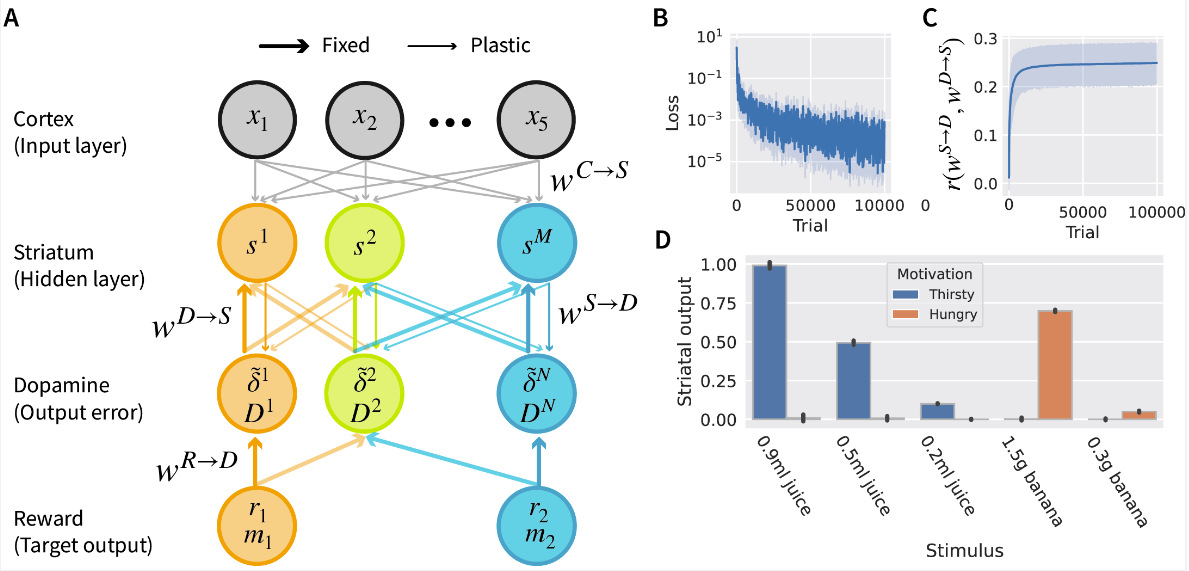

On a related note, more care should be taken to accurately compare this work to ref. [45] and the capabilities of homeostatic RL.

↪ We extended the comparison with homeostatic RL and added description of capabilities of this model:

> "··· *While the Reward Bases model defines the reward as the linear summation of elementary rewards scaled by their motivational needs, the homeostatic RL defines the reward as a reduction in drive and defines drive as a nonlinear combination of different dimensions of the distance between the current and desired physiological state. Consequently, homeostatic RL can account for multiple experimentally observed phenomena, for example that deprivation of one need reduces the rewarding value of other outcomes [45].* ···" — *Page 19, Lines 581-586*

We call the authors attention to: Duriez, Alexia, et al. "Homeostatic reinforcement theory accounts for sodium appetitive state-and taste-dependent dopamine responding." Nutrients 15.4 (2023): 1015. This paper is also relevant to the authors' work and merits discussion in the context of the submitted manuscript.

↪ We have added a discussion of this study:

> "··· *The homeostatic RL model has also been applied to explain behaviour in tasks where animals were repeatedly offered choice between salt solutions, and to explain dopamine responses to salt infusion [90].* ···"
> — *Page 19, Lines 586-588*

Reviewer 2

In the original review I pointed out that the models of the data on dopamine coding of errors in different dimensions (pg. 9) had an inferential error by violating the hierarchical principle. Specifically the model with a main effect of value and an interaction term for value and stimulus Identity was selected as the "best" fit, even though the main effect term of stimulus identity was not included in the model. Indeed, as the authors point out in their reply that the correct form of this model (with both main effects along with the interaction term) provides a substantially worse fit to the data than the incorrect model (and even worse than the stimulus only model) (Fig. 4A). Suggesting that, in the space of these feature sets, the model they infer from is not, in fact, the best model.

The authors reply that this incorrect form of the model is equivalent to a later model including the main effect of food prediction error and the main effect of juice prediction error. I've spent the better part of today trying to work through that equivalence proof, but to no luck (admittedly I'm not a very good mathematician).

So I have few lingering concerns here:

Even though this model is later shown to be equivalent to a main effects only model, in the space of features being evaluated in this set of comparisons, it is not in fact a winning model because it is not a valid form of the model. The correct form of the model is indistinguishable (or worse than) a stimulus only model, making the inference from *these* feature comparisons ambiguous at best. Could the authors provide more details on the equivalence proof for these two models? This is a critical inference being made to justify the reward bases model and if I'm getting stuck on the equivalence justification, I have a feeling many other readers might get stuck as well. I get what the authors are trying to do here by building up to their model, but it seems really odd to have a feature test comparison where the model that "wins" the comparison tests is statistically ill-formed (i.e., violates the hierarchical principle), but later shown to be equivalent to a model that is properly formed (i.e., a simple main effect model with different features). The reader is asked to hold a contradiction in their head until later (or read very very carefully and work out a proof on their own). So now I am confused why the versions of the models run in this section (and shown in Figure 4) are relevant if the goal is to just get to the reward bases model. Why not just jump straight into a reward bases model?

↪ We thank the Reviewer for this valuable suggestion. We changed the order of presentation of regression models in Section 2.3, and first present the model equivalent to Reward Bases (with juice and banana regressors), which is most intuitive and does not include any interactions. Only afterwards we present a series of regression models based on reward and identity regressors, as a mean to quantify statistically if the the activity of dopamine neurons depends on reward identity.

Additionally, we extended the explanation of the equivalence between the two sets of regression models, and added an equation demonstrating the equivalence explicitly:

> "··· *We now show that the regression model of Equation 15 is equivalent to that of Equation 12. We first note that there exist relationships between regressors in the two models:* $r_1(x) = R(x)(I(x)+1)/2$ *and* $r_2(x) = R(x)(-I(x)+1)/2$. *For example, if x is one of the stimuli predicting juice, then* $I(x) = 1$, *so* $R(x)(I(x)+1)/2 = R(x) = r_1(x)$. *It is insightful to substitute these relationships into Equation 12:* ···" — *Page 23, Lines 763-766*

$$
\begin{aligned}
S &= \beta_1^k R(x)(I(x)+1)/2 + \beta_2^k R(x)(-I(x)+1)/2 + \beta_0^k + \omega \\
&= (\beta_1^k + \beta_2^k)/2 \ R(x) + (\beta_1^k - \beta_2^k)/2 \ R(x)I(x) + \beta_0^k + \omega
\end{aligned}
$$

We have also modified the figure showing the regression analyses so it presents the regression model equivalent to Reward Bases first (in panel A):

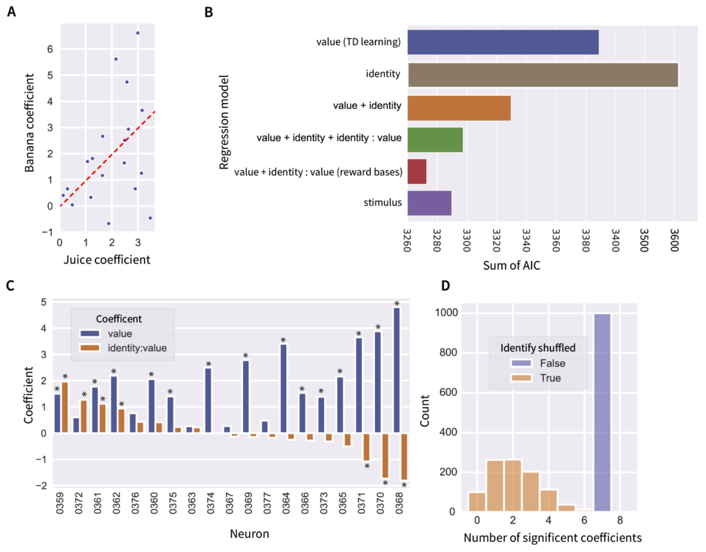

To make models in panel B of the above figure easier to identify, we indicated in brackets which regression models correspond to TD-learning and Reward Bases.
